# A novel m.14677 T > C variant in mitochondrial *tRNA*^*Glu*^ gene causes chronic progressive external ophthalmoplegia

**DOI:** 10.1038/s10038-025-01381-7

**Published:** 2025-08-06

**Authors:** Nahoko Katayama Ueda, Masakazu Mimaki, Shota Ito, Ayuka Murakami, Satoshi Yokoi, Ichizo Nishino, Masahisa Katsuno, Yu-ichi Goto

**Affiliations:** 1 Medical Genome Center, National Institute of Neuroscience, National Center of Neurology and Psychiatry, Tokyo, Japan; 2 Department of Pediatrics, Graduate School of Medicine, The University of Tokyo, Tokyo, Japan; 3 Department of Pediatrics, Teikyo University School of Medicine, Tokyo, Japan; 4 Department of Neurology, Nagoya University Graduate School of Medicine, Aichi, Japan; 5 Department of Pathophysiological Laboratory Sciences, Nagoya University Graduate School of Medicine, Aichi, Japan; 6 Department of Neuromuscular Research, National Institute of Neuroscience, National Center of Neurology and Psychiatry, Tokyo, Japan

**Keywords:** Genetics research, Disease genetics

## Abstract

Chronic progressive external ophthalmoplegia (CPEO) is a mitochondrial disease characterized by progressive ptosis and ophthalmoplegia, caused by single deletions, point mutations, or multiple deletions in mitochondrial DNA (mtDNA). Most point mutations occur in tRNA genes. Here, we report a novel variant of the *tRNA*^*Glu*^ gene associated with CPEO. A 45-year-old male presented with ptosis and external ophthalmoplegia; however, blood test results, including lactate levels and autoantibodies, were normal. CPEO was suspended, prompting additional myopathological examination, mtDNA sequencing analysis, long polymerase chain reaction (PCR) analysis, and single-fiber analysis to compare mutation loads between ragged-red fibers (RRFs) and non-RRFs. Histopathological examination revealed scattered COX-negative RRFs. No deletions were found in the mtDNA. MtDNA sequencing analysis revealed a novel variant, m.14677 T > C, in the *tRNA*^*Glu*^ gene, with Sanger sequencing indicating 45% heteroplasmy in the muscle tissue. Single-fiber analysis showed a significantly higher mutation load of m.14677 T > C in RRFs (range: 25.3–92.8%; median: 88.1%; *n* = 6) compared with non-RRFs (range: 3.5–85.9%; median: 17.1%; *n* = 5) (*P* = 0.03). Based on the significantly higher mutation load in RRFs than in non-RRFs, pathological evidence of mitochondrial disease, and the mutation’s occurrence at an evolutionarily conserved site, we concluded that m.14677 T > C, a novel variant of the *tRNA*^*Glu*^ gene, is the cause of CPEO. Biochemical and histopathological examinations of muscle tissue, combined with single-fiber analysis, are valuable tools for evaluating mtDNA variants, particularly those within tRNA genes.

## Introduction

Chronic progressive external ophthalmoplegia (CPEO) is one of the most common mitochondrial diseases, characterized by progressive ptosis and ophthalmoplegia. The most common cause of CPEO is a single large deletion of mitochondrial DNA (mtDNA), accounting for approximately 60% of cases [1, 2]. Other causes include multiple deletions of mtDNA due to mutations in nuclear genes and mtDNA point mutations.

Human mtDNA encodes 22 different transfer RNAs (tRNAs) essential for translating 13 different mtDNA-encoded proteins. Point mutations in (mtDNA) point mutations that cause CPEO predominantly occur in tRNA genes. Although tRNA genes constitute only a small portion of the mitochondrial genome, tRNA genes are hotspots for mitochondrial point mutations, including m.3243 A > G, which is associated with mitochondrial encephalopathy, lactic acidosis, and stroke-like episodes (MELAS). To date, more than 750 pathogenic mtDNA mutations have been identified, with 300 ocurring in specific tRNA genes [3].

In this study, we performed a single-fiber analysis of a novel variant, m.14677 T > C, within the *tRNA*^*Glu*^ gene to confirm its pathogenicity.

## Materials and methods

### Patient’s history and clinical findings

**Fig. 1:**
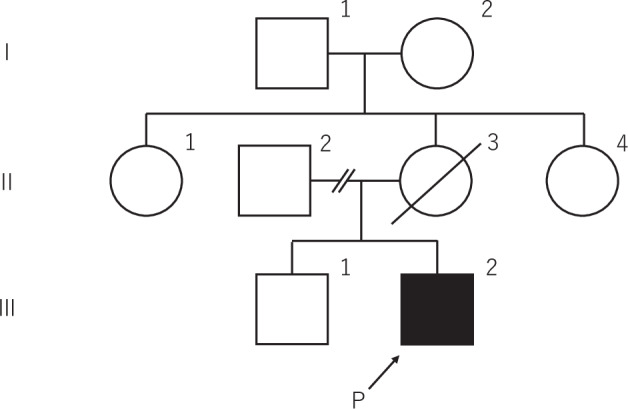
Pedigree of the family. The proband is indicated by an arrow

The patient was a 45-year-old Japanese male with no relevant medical history. He had no family history of similar symptoms, although his mother (Fig. 1 II-3) died at the age of 30 years due to collagen disease.

The patient reported awareness of diplopia since the age of 42, with worsening symptoms leading him to seek medical care at the age of 45. Clinical examination revealed bilateral ptosis and ophthalmoplegia, without diurnal changes. No muscle weakness, sensory symptoms, signs of ataxia, or retinal abnormalities were observed. Blood tests showed normal lactate levels, and other parameters, including creatine kinase levels, were within normal limits. Tests for anti-acetylcholine receptor and anti-muscle-specific tyrosine kinase antibodies were negative. Whole-body computed tomography (CT) scan showed no evidence of muscle atrophy, and the electrocardiogram was normal.

Based on these clinical findings, CPEO was suspected. To confirm the diagnosis, a muscle biopsy of the left biceps brachii muscle was performed. Histopathological examination, mtDNA sequencing analysis, long polymerase chain reaction (PCR) analysis using DNA extracted from the muscle, and single-fiber analysis of muscle fibers were subsequently conducted.

### Histopathological examination

Ten-μm-thick transversely oriented serial cross-sections of frozen muscle were stained with hematoxylin and eosin (H&E), modified Gomori trichrome (mGT), and a double stain of cytochrome *c* oxidase (COX)/succinate dehydrogenase (SDH) (COX/SDH staining) to detect ragged-red fibers (RRFs). Pathological diagnoses were made using an optical microscope (OM).

### Mitochondrial DNA analysis

#### Long PCR

Total genomic DNA was extracted from the skeletal muscle and blood using standard procedures. The mtDNA was amplified in two fragments using long PCR with Platinum SuperFi DNA Polymerase (Thermo Fisher Scientific K.K., Tokyo, Japan). Long PCR was performed to exclude mtDNA- like sequences in the nuclear DNA [4] and to detect mtDNA rearrangements. The primers used for long PCR are listed in Supplementary Table 1.

#### Sequencing analysis

Long PCR products were purified using the Agencourt AMPure XP Kit (Nippon Genetics, Tokyo, Japan) and sequenced using the MiSeq Reagent Kit v2 with a 2 $\times$ 150 bp run on a sequencer (MiSeq system, Illumina K.K., Tokyo, Japan). Data were aligned to the revised Cambridge reference sequence for human mtDNA (GenBank Accession number: NC_012920.1) and analyzed using MiSeq Reporter software (Illumina K.K., Tokyo, Japan). Candidate pathogenic variants were confirmed through Sanger sequencing.

#### Pyrosequencing

The mutation rate of candidate pathogenic variants in mtDNA extracted from the muscle and the blood were also confirmed by pyrosequencing using PyroMark Q24 Advanced (Qiagen K.K., Tokyo, Japan). The primers used for pyrosequencing are listed in Supplementary Table 2.

### Single-fiber analysis

Laser capture microdissection was performed on 10-µm thick SDH-stained muscle sections to isolate individual muscle fibers (RRFs and non-RRFs) using LMD-7 system (Leica Microsystems, Tokyo, Japan). Ten RRFs and ten non-RRFs were isolated and mutational loads were confirmed by pyrosequencing. For the first PCR, 16 µL of distilled water was added to the tubes containing the laser-microdissected sections, and the first PCR was performed. PCR products were purified using a FastGene Gel/PCR Extraction Kit (Nippon Genetics, Tokyo, Japan). Half of each sample was used as a template for the second round of PCR, which was performed twice to confirm reproducibility.

### Approval of the ethics review committee

This study was approved by the Research Ethics Committee of the National Centre of Neurology and Psychiatry (Approval number: A2019-123).

## Results

### Histopathological findings

**Fig. 2:**
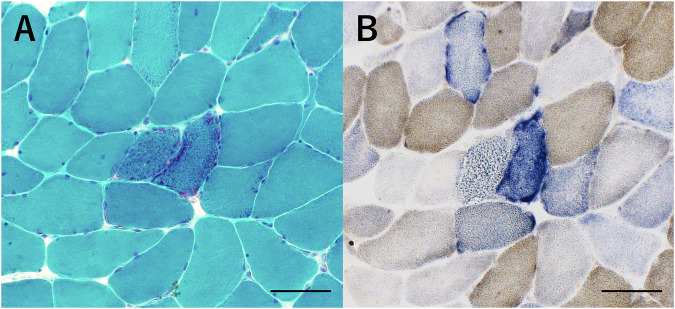
Representative muscle pathology. Bar = 50 µm. **A** Modified Gomori trichome (mGT) staining showed several ragged-red fibers (RRFs). **B** Cytochrome c oxidase (COX)/succinate dehydrogenase (SDH) (SDH/COX) staining showed COX-negative RRFs

H&E staining revealed mild to moderate variations in fiber size. The mGT staining identified several RRFs and pathological accumulations indicative of abnormal mitochondrial proliferation. For the SDH/COX, most RRFs were COX-negative; however, a few displayed COX variability. No strongly SDH-reactive blood vessels were observed. (Fig. 2A, B)

### Mitochondrial DNA analysis

**Fig. 3:**
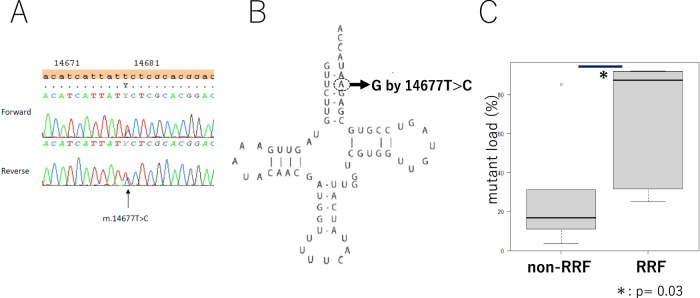
**A** A novel heteroplasmic variant, m.14677 T > C, in the *tRNA*^*Glu*^ gene, which was confirmed by Sanger sequencing. **B** Molecular location of the m.14677 T > C variant. Nucleotide position 14,677 is located in the aminoacyl acceptor stem of *tRNA*^*Glu*^. **C** A statistically significant higher mutation load of the m.14677 T > C variant in RRFs (range: 25.3–92.8%; median: 88.1%; *n* = 6) compared with non-RRFs (range: 3.5–85.9%; median: 17.1%; *n* = 5); *P* = 0.03

Next-generation sequencing (NGS) of the complete mtDNA sequence from the patient’s muscle sample revealed a novel heteroplasmic variant, m.14677 T > C, in the *tRNA*^*Glu*^ gene, which was confirmed by Sanger sequencing (Fig. 3A, B). The proportion of mutant mtDNA in the muscle samples was 43.1% and 9.3% in the blood samples, respectively. Long PCR analysis revealed only full-length products, with no evidence of mitochondrial rearrangement.

### Single-fiber analysis

Ten samples were prepared from RRFs and non-RRFs. First PCR products were obtained from six RRFs and five non-RRFs. Single-fiber analysis of individual RRFs and non-RRFs revealed significantly higher mutation loads of the m.14677 T > C variant in RRFs (range: 25.3–92.8%; median: 88.1%; *n* = 6) compared with non-RRFs (range: 3.5–85.9%; median: 17.1%; *n* = 5; *P* = 0.03) (Fig. 3C).

## Discussion

In this study, we identified a novel heteroplasmic m.14677 T > C variant in the *tRNA*^*Glu*^ gene in a Japanese male patient who presented with CPEO. This variant was located in the aminoacyl acceptor stem of *tRNA*^*Glu*^ (Fig. 3B). Several mutations have been reported within the tRNA^Glu^ gene, including those associated with infantile reversible respiratory chain deficiency, encephalopathy, and CPEO [5, 6] (Supplementary Table 3). The phenotypes of *tRNA*^*Glu*^ gene mutations are heterogeneous, a characteristic also observed in other mt-tRNA genes.

The pathogenicity of the variant was predicted using MITOTIP, yielding a score of 73.1% [3]. Single-fiber analysis is a reliable method for evaluating the pathogenicity of tRNA variants [7, 8]. It is well-established that demonstrating a higher mutation load in pathologically abnormal muscle fibers through single-fiber analysis provides strong evidence for the pathogenicity of tRNA variants. In this study, single-fiber analysis revealed a significantly higher mutation load of the novel m.14677 T > C variant in RRFs, confirming its pathogenicity. In addition, the lower mutation load in blood compared to muscle, suggesting tissue specificity, also supports its pathogenicity.

Criteria incorporating single-fiber analysis to assess the pathogenicity of tRNA variants were proposed by DiMauro et al. and McFarland et al., as shown in Tables 1, 2, respectively [9, 10]. Using this criterion, we evaluated the pathogenicity of the m.14677 T > C variant. The base changes in this mutation occurred at evolutionarily conserved sites (Table 3). Most of the criteria described by DiMauro et al. were met, and the criteria proposed by McFarland et al. classified the variant as probably pathogenic, with a score of 11 points. Overall, these findings strongly indicate that the m.14677 T > C variant in the *tRNA*^*Glu*^ gene is the causative mutation of CPEO in the present case.

**Table 1:** Comparison of DiMauro et al. criteria with the present case, adapted from Ref. [9]

DiMauro’s criteria	Present case
The mutation must not be a known neutral polymorphism.	〇
The base change must affect an evolutionarily conserved and functionally important site.	〇 See Table 3
Deleterious mutations are usually heteroplasmic.	〇
The degree of heteroplasmy in different family members ought to be in rough agreement with the severity of symptoms.	Unknown since this is the first case.
Single-fiber PCR, demonstrating higher levels of mutation in abnormal fibers such as RRF.	〇

**Table 2:** Comparison of Mcfarland et al. criteria with the present case, adapted from Ref. [10]

Mcfarland’s criteria	Present case	Score
Evolutionary conservation of the base	〇 See Table 3	2
More than one independent report	〇	2
Presence of heteroplasmy	〇	2
Histochemical evidence of mitochondrial disease	〇	2
Biochemical defect in complexes I, III or IV	uninspected	0
Segregation of the mutation with disease	uninspected	0
Single-fiber studies, demonstrating higher levels of mutation in COX negative fibers	〇	3
Steady-state levels of mutated mt-tRNA or evidence of pathogenicity from cybrid cells	uninspected	0

≥14 points: Definitely pathogenic; 11–13 points: Probably pathogenic; 7–10 points: Possibly pathogenic; ≤6 points: Neutral variants

Total: 11 points = Probably pathogenic

**Table 3:** The m.14677 T > C variant occurs at an evolutionary conserved site in the mitochondrial genome, with a conservation rate of 100% in primates and 97.78% in all species [3]

Species							
Patient	T	A	T	**C**	C	T	C
Homo sapiens (rCRS)	T	A	T	**T**	C	T	C
Pan troglodytes	T	A	T	**T**	C	T	C
Gorilla gorilla	C	A	T	**T**	C	T	C
Bos taurus	T	A	T	**T**	C	T	T
Felis catus	T	A	T	**T**	C	T	C
Rattus norvegicus	T	A	T	**T**	T	C	T
Gallus gallus	T	A	T	**T**	C	C	C
Cyprinus carpio	A	A	T	**T**	C	T	T
Xenopus laevis	A	A	T	**T**	C	C	C
Alligator mississippiensis	T	A	T	**T**	T	T	T
Drosophila melanogaster	-	-	A	**A**	T	T	T

In conclusion, we demonstrated that the novel m.14677 T > C variant in the *tRNA*^*Glu*^ gene is responsible for CPEO, as confirmed through single-fiber analysis. Biochemical and histopathological examinations of muscle tissues, combined with single-fiber analysis, are valuable tools for evaluating mtDNA variants, especially those within tRNA genes.

## Supplementary information

Supplementary Tables
