# Supplementary material for: A novel m.14677 T > C variant in mitochondrial tRNAGlu gene causes chronic progressive external ophthalmoplegia
Source: J Hum Genet. 2025 Aug 6;70(10):537–40. doi: 10.1038/s10038-025-01381-7 (PMC12460166; doi:10.1038/s10038-025-01381-7)
Supplement: Supplementary file 1 — Supplementary Tables [file 10038_2025_1381_MOESM1_ESM.docx]

**Supplementary Tables**

**Supplementary Table 1.** Primers used for long polymerase chain reaction (PCR).

| Long fragment, m.5200_m.16427 (11,228 bp) | Short fragment, m.15756_m.5739 (6,553 bp) |
| --- | --- |
| Forward: 5’-TTC CAT CCA CCC TCC TCT CCC TAG-3’ | Forward: 5’-GAA TCG GAG GAC AAC CAG TA-3’ |
| Reverse: 5’-GGG ATA TTG ATT TCA CGG AGG ATG G-3’ | Reverse: 5’-GCG GGA GAA GTA GAT TGA AG-3’ |

**Supplementary Table 2.** Primers used for pyrosequencing.

| **First PCR primer** | **Second PCR primer** | **Sequencing primer** |
| --- | --- | --- |
| Forward: 5’-ATT CAG CTT CCT ACA CTA TT-3’ | Forward: 5’-CTA AAC CCA CAC TCA ACA GAA ACA-3’ | Forward, 5’-GAA ACA AAG CAT ATA TCA T-3’ |
| Reverse: 5’- TTC ATC ATG CGG AGA TGT TG-3’ | Reverse: 5’-TTA TTA GGG GGT TAA TTT TGC GT-3’ |  |

**Supplementary Table 3.** Previously reported pathological variants in the MT-tRNAglu gene. [3]

| Pathogenic variant | Reported clinical phenotype |
| --- | --- |
| m.14674T>C | Reversible COX deficiency |
| m.14677T>C | This patient |
| m.14709T>C | Mitochondrial myopathy, Diabetes / Encephalomyopathy / Deafness, Mental retardation, Cerebellar dysfunction |
